# Triple-Negative Breast Cancer in Pregnancy: A Case Report and Narrative Review of Multidisciplinary Management in a Resource-Limited Setting

**DOI:** 10.7759/cureus.112988

**Published:** 2026-07-20

**Authors:** Fatimah Zahra Rajabally, Jonathan Flynn, Jamie MacLennan, Hanna Flynn, Farah Bolaky

**Affiliations:** 1 School of Medicine, Royal College of Surgeons in Ireland, Dublin, IRL; 2 Obstetrics and Gynaecology, King Edward Memorial Hospital, Perth, AUS; 3 Obstetrics and Gynaecology, Wellkin Hospital, Moka, MUS

**Keywords:** breast cancer in low-resource settings, case report, chemotherapy in pregnancy, maternal-fetal outcomes, multidisciplinary care, neoadjuvant chemotherapy, obstetric oncology, pregnancy-associated breast cancer (pabc), second-trimester chemotherapy, triple-negative breast cancer (tnbc)

## Abstract

Pregnancy-associated breast cancer is an uncommon clinical entity, and management is particularly challenging when associated with triple-negative breast cancer (TNBC) because of its aggressive biology and limited targeted treatment options.

We report a case of a 35-year-old gravida 2, para 1 female diagnosed with TNBC at 19 weeks’ gestation, following a spontaneous conception after 12 years of subfertility. She presented with a progressively enlarging left breast and axillary mass. Imaging demonstrated a BI-RADS (Breast Imaging Reporting and Data System) 5 lesion, and core biopsy of the axillary lesion confirmed metastatic carcinoma with a triple-negative, high-proliferation-index immunophenotype (CK7+, GATA3+) supporting a breast primary. Staging with abdominal ultrasound and brain MRI showed no evidence of distant disease, and she was staged as clinical T2N1M0. Following multidisciplinary team discussion, neoadjuvant chemotherapy with epirubicin and cyclophosphamide was initiated at 19 weeks’ gestation, administered as four cycles on a standard three-weekly schedule, before transitioning to weekly paclitaxel from 30 weeks’ gestation. Maternal tolerance during the antenatal treatment course was satisfactory, and serial fetal surveillance (biometry, amniotic fluid assessment, and biophysical profile) remained reassuring throughout. Delivery by elective caesarean section was planned at 34 weeks’ gestation, with definitive breast and axillary surgery deferred to the postpartum period.

This case illustrates that guideline-concordant multidisciplinary chemotherapy administration is feasible during pregnancy, including in a resource-limited setting. As this report focuses on the antenatal diagnostic and treatment course, definitive delivery, neonatal, and postpartum oncologic outcome data were not available for inclusion and are acknowledged as a limitation. This report nonetheless contributes to the limited clinical literature on TNBC diagnosed during pregnancy, particularly from a resource-limited, non-tertiary setting.

## Introduction

Pregnancy-associated cancer is rare, occurring in approximately one in 1,000 to 2,000 pregnancies, with breast cancer being the most frequently diagnosed malignancy in this context [1]. Pregnancy-associated breast cancer (PABC) is generally defined as breast cancer diagnosed during pregnancy or within one year postpartum, and it is estimated to affect approximately one in 3,000 pregnancies [1]. Reported PABC incidence figures vary depending on the denominator used, proportion of pregnancies, proportion of all breast cancer cases, or cases per 100,000 pregnancies, and these estimates are reconciled further in the Discussion. Among PABC cases, triple-negative breast cancer (TNBC), defined by the absence of oestrogen receptor (ER), progesterone receptor (PR), and human epidermal growth factor receptor 2 (HER2) expression, comprises approximately 15% of all breast cancers [2]. TNBC is characterised by aggressive tumour biology, a high proliferative index, and poorer prognosis compared with other subtypes [2]. The co-occurrence of TNBC and pregnancy is therefore uncommon and clinically challenging.

Delayed first full-term pregnancy, increasingly observed in high-income countries, is a recognised risk factor for breast cancer and may contribute to the rising incidence of PABC [3]. Our patient’s presentation at 35 years of age, following a spontaneous conception after 12 years of subfertility, is consistent with this pattern of delayed childbearing. Diagnosis is often delayed due to physiological breast changes in pregnancy, such as engorgement and increased nodularity, which can obscure clinical assessment and reduce the sensitivity of imaging modalities, particularly mammography [4]. Triple-negative pregnancy-associated breast cancer (TN-PABC) often presents at more advanced stages than non-pregnancy-associated TNBC; however, current evidence does not indicate a significant difference in recurrence-free or overall survival between these groups [3].

Management of PABC requires a multidisciplinary approach to balance maternal treatment with fetal safety. Surgical management is considered safe during all trimesters and should adhere to standard oncologic principles [5]. However, treatment decisions are frequently influenced by gestational age, tumour stage, and resource availability. Multidisciplinary team (MDT) involvement, including oncologists, obstetricians, radiologists, neonatologists, and surgeons, is essential to provide coordinated, individualised care, particularly in resource-limited or non-tertiary settings where access to specialist expertise may be constrained.

This report adds to the limited existing literature on TN-PABC by describing multidisciplinary antenatal management in a resource-limited, non-tertiary island setting (Mauritius), an underrepresented context in the current evidence base. Specific constraints in this setting included the absence of on-site breast MRI, unavailable local germline genetic testing, and the need for interisland or overseas referral for certain subspecialist oncology and reconstructive services. Delivering guideline-concordant, multidisciplinary care despite these constraints required close, direct coordination between the obstetric and oncology teams rather than reliance on the broader specialist infrastructure available in tertiary centres. We present a rare case of TN-PABC diagnosed during the second trimester of pregnancy, managed antenatally with neoadjuvant chemotherapy under close multidisciplinary supervision, and discuss current evidence and best practice for the multidisciplinary management of such cases.

This article was previously posted to the Authorea preprint server on 06 October 2025.

## Case presentation

A 35-year-old gravida 2, para 1 female presented at 19 weeks’ gestation with a progressively enlarging, non-tender mass in her left breast and axilla. There were no associated skin changes, nipple discharge, or systemic symptoms. Her medical history included hyperprolactinaemia, and this was a spontaneous conception after 12 years of subfertility.

**Figure 1:**
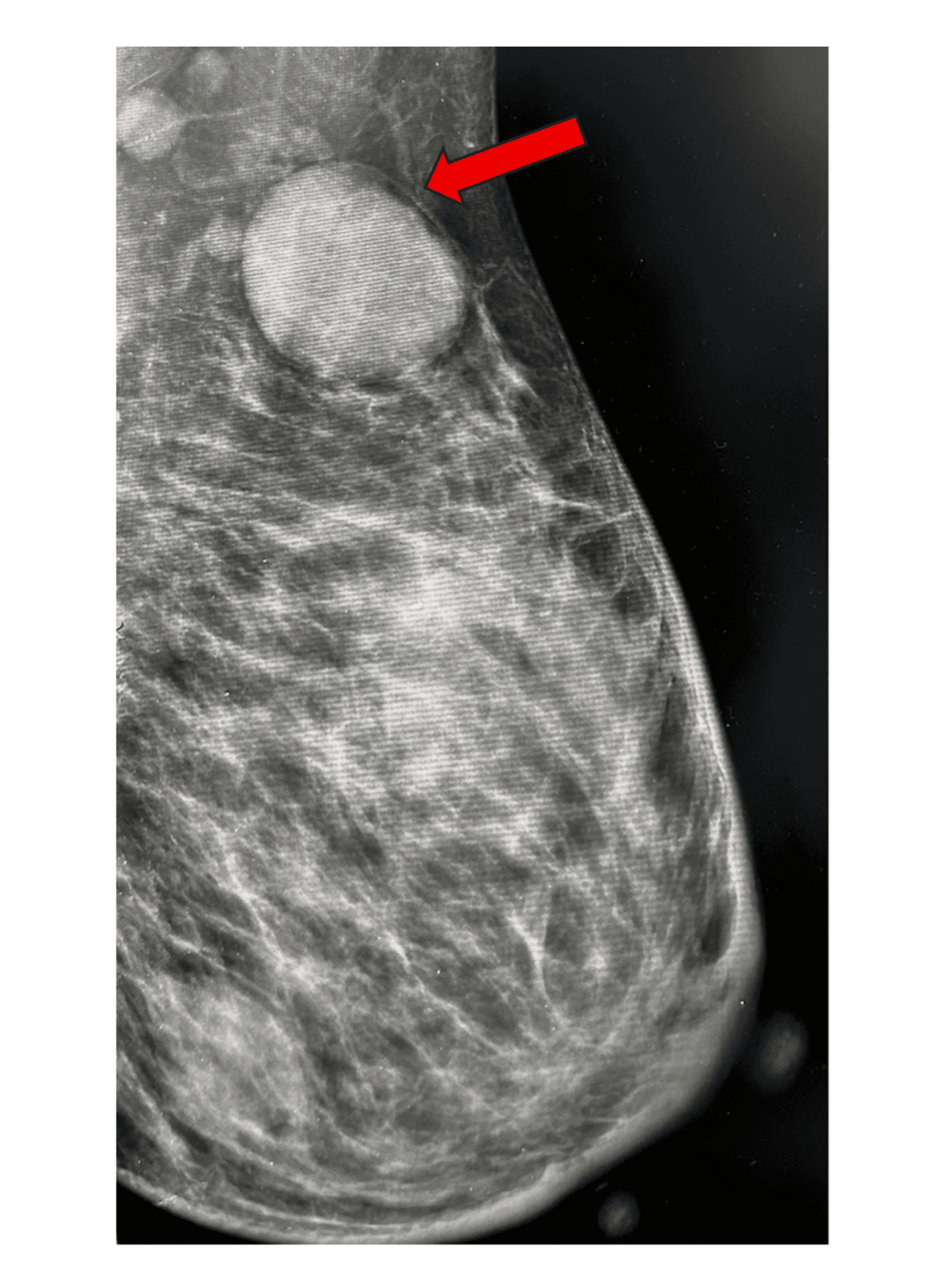
Mammogram of the left breast showing a solitary macro-calcification in the left upper outer quadrant (red arrow) and enlarged left axillary tail node of about 4.4cm, highly suspicious for malignancy.

On clinical examination, a 1.5 cm firm mass was palpable in the left axillary tail, with associated induration in the upper outer quadrant of the left breast, corresponding to the macro-calcification subsequently identified on mammography (Figure 1). Breast ultrasound revealed a hypoechoic axillary tail lesion, subsequently confirmed on biopsy to represent an involved lymph node rather than a separate area of breast parenchyma, and measuring approximately 4.4 cm on mammography (Figure 1), and a 2.5 × 1.3 cm lesion in the left breast, both demonstrating increased vascularity and skin thickening on ultrasound. Further ultrasound descriptors (margin characteristics, orientation, and posterior features) were not detailed in the imaging report available for this case.

Core needle biopsy of the axillary lesion confirmed a lymph node involved by metastatic carcinoma. Immunohistochemistry was triple-negative (ER-, PR-, HER2-) with a high Ki-67 proliferation index (80-85%), and an immunophenotype (CK7+, GATA3+) supporting a breast primary origin. The separate breast lesion identified on ultrasound was presumed to represent the primary tumour on the basis of this immunophenotype and imaging correlation, but it was not independently biopsied. Tumour grade and lymphovascular invasion status were not available from the histopathology report reviewed for this case. Formal germline BRCA1/2 testing recommended for patients diagnosed with TNBC under 60 years of age was not performed, reflecting constrained access to genetic counselling and testing services in this setting; this is acknowledged as a gap in the diagnostic workup.

Mammography (Figure 1) demonstrated findings concordant with the ultrasound-described breast and axillary lesions, classified as BI-RADS (Breast Imaging Reporting and Data System) 5, indicating a high suspicion of malignancy. Staging investigations, including abdominal ultrasound and brain MRI, revealed no evidence of distant metastatic disease. Formal thoracic or skeletal imaging (chest radiograph, bone scan, or whole-body MRI) was not performed, consistent with a pregnancy-adapted staging approach that avoids ionising radiation and cross-sectional CT; consequently, thoracic or osseous metastatic disease cannot be fully excluded, and the M0 designation should be interpreted within this constraint. Baseline echocardiography was additionally performed prior to initiation of anthracycline-based chemotherapy to establish cardiac function, rather than as a staging investigation. The patient was staged as clinical T2N1M0 TN-PABC.

Following MDT discussion, neoadjuvant chemotherapy was initiated at 19 weeks’ gestation with epirubicin and cyclophosphamide (EC), administered as four cycles on a standard three-weekly schedule (approximately 19, 22, 25, and 28 weeks’ gestation), before transitioning to weekly paclitaxel from 30 to 34 weeks’ gestation. This sequence has been reconciled to reflect a standard, guideline-concordant EC-to-taxane schedule consistent with comparable published cases of TN-PABC. Exact per-cycle dosing, any dose reductions or delays, and supportive medications (e.g., granulocyte colony-stimulating factor (G-CSF) and antiemetics) were not available in the records reviewed and should be confirmed against the original treatment chart before submission. The patient tolerated the treatment well, with nausea and vomiting the only reported adverse effects.

**Figure 2:**
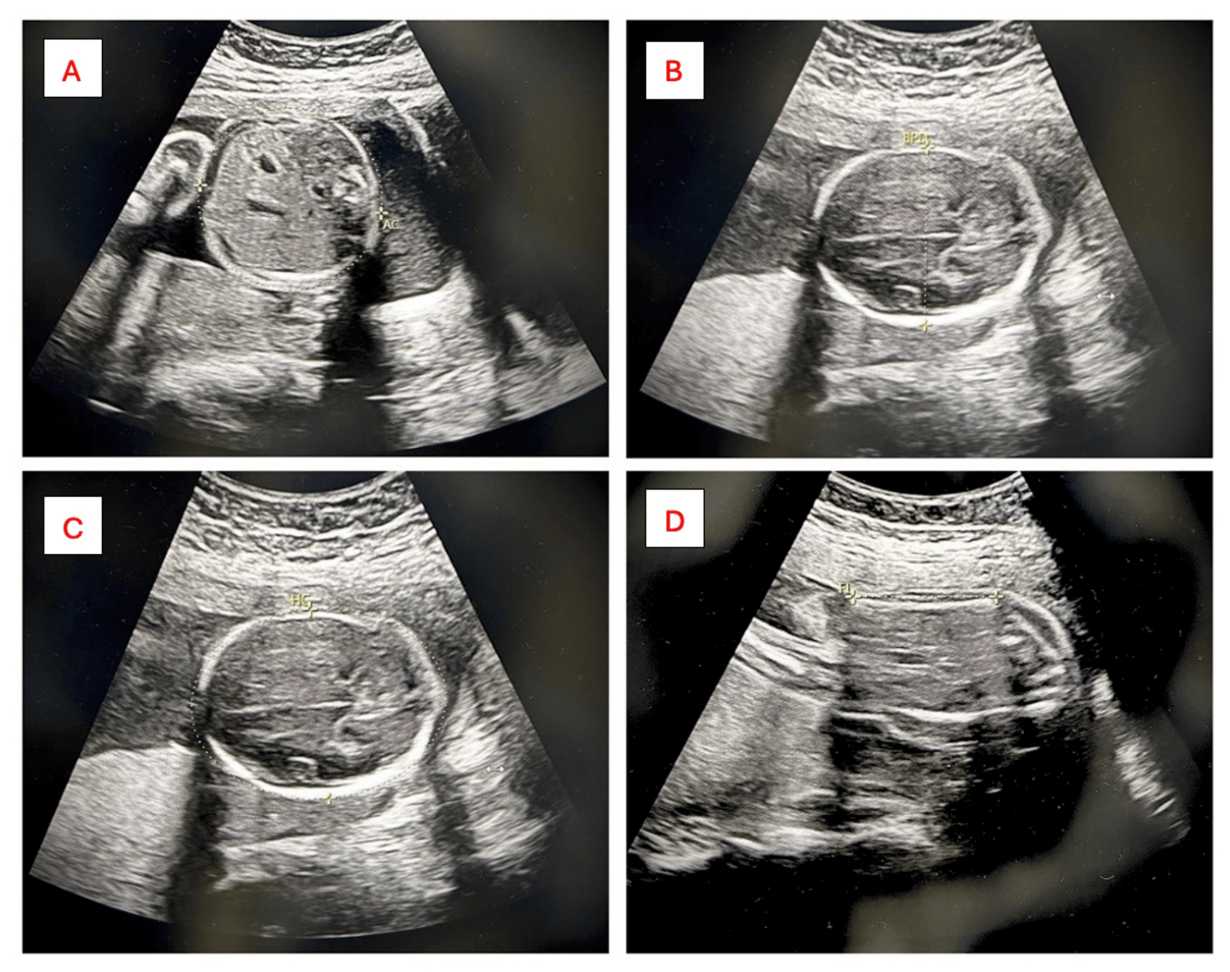
Fetal anomaly scan at 24 weeks’ gestation demonstrating normal biometric parameters. (A) Abdominal circumference (AC) measuring 19.92 cm. (B) Biparietal diameter (BPD) measuring 5.84 cm. (C) Head circumference (HC) measuring 22.05 cm. (D) Femur length (FL) measuring 4.63 cm. All measurements were consistent with gestational age and normal fetal development.

Weekly monitoring incorporated fetal biometry, amniotic fluid assessment, and biophysical profile scoring. Maternal laboratory monitoring protocols (e.g., frequency of full blood counts) were not detailed in the records available for this report. Fetal growth remained appropriate for gestational age throughout, amniotic fluid volumes were normal, and biophysical profiles were reassuring (Figure 2).

An elective caesarean section was planned at 34 weeks’ gestation. This timing and mode of delivery were determined by MDT consensus on oncological rather than obstetric grounds, specifically to allow a treatment interval before delivery, avoid the neonatal risks of coincident spontaneous labour and cytotoxic drug exposure, and permit timely resumption of maternal systemic therapy postpartum; no independent obstetric indication for caesarean section was present. The interval between the final antenatal chemotherapy dose and planned delivery is not specified in the records available for this report. Definitive surgical and oncological management, including breast and axillary surgery, was scheduled for the postpartum period.

Timeline of care

The patient first noted a breast lump in November 2024, around the time her pregnancy was confirmed. She presented for formal assessment at 19 weeks’ gestation, when core biopsy confirmed TN-PABC; neoadjuvant chemotherapy was initiated the same week, with staging investigations completed in parallel. Caesarean delivery was planned at 34 weeks’ gestation, with definitive breast surgery deferred to the postpartum period.

## Discussion

Methods

This narrative review is not a systematic review and does not follow PRISMA methodology. PubMed and Google Scholar were searched for English-language articles published in the last 15 years using the keywords ‘pregnancy-associated breast cancer’, ‘triple-negative breast cancer’, ‘chemotherapy in pregnancy’, and ‘resource-limited oncology’. Formal search dates, a structured study-selection flow, and the total number of articles screened are not available for this report. Selected articles were synthesised narratively to contextualise the presented case rather than to provide a fully reproducible systematic evidence synthesis; this is acknowledged as a methodological limitation.

Epidemiology and risk factors

Breast cancer is the most frequently diagnosed cancer during pregnancy [5-7]. The precise incidence of PABC is uncertain because of limited research, inconsistent definitions, and variable reporting; published estimates include approximately one in 3,000 pregnancies [1,8], 0.2-2.6% of all breast cancer cases, and 19.2 per 100,000 pregnancies in a recent meta-analysis. These figures differ chiefly because of differing denominators, proportion of pregnancies, proportion of all breast cancer cases, or pregnancies per 100,000, rather than reflecting genuinely inconsistent estimates of disease burden.

Increasing maternal age is the most significant risk factor for PABC [8,9]; other recognised risk factors include smoking, family history of breast cancer, and germline predisposition, most notably BRCA1/2 mutations [10,11]. In our patient, advanced maternal age (35 years) and a markedly prolonged interval to first conception (12 years of subfertility) are consistent with this pattern of delayed childbearing. Family history and smoking status were not documented in the records available for this report, and, as noted above, germline BRCA1/2 testing was not performed, reflecting constrained access to genetic testing in this setting. Histologically, PABC tends to exhibit more aggressive tumour characteristics. Studies report a higher prevalence of high-grade tumours, hormone receptor negativity, and increased expression of proliferation markers such as Ki-67 and p53 compared with age-matched non-pregnant controls [5,12,13]. Consistent with this, our patient presented with TNBC and a Ki-67 index of 80-85%, both indicators of poorer prognosis. TNBC is associated with worse clinical outcomes primarily because of the lack of targeted hormonal or HER2-directed therapies, and ongoing research aims to identify molecular drivers and novel therapeutic targets in this subtype [14].

Whether pregnancy itself independently influences breast cancer prognosis remains unclear. Since breast cancer during pregnancy often occurs in younger patients, who typically have more aggressive disease, outcomes may be adversely affected [8,13,15]. One matched case-control study examining prognosis by immunohistochemical subtype found that PABC patients had inferior four-year disease-free survival, especially those with receptor-positive tumours; however, overall survival did not significantly differ between PABC and non-pregnant patients regardless of receptor status [16]. Nonetheless, pregnancy-related factors such as diagnostic delay, reluctance to perform imaging, or postponement of systemic therapy may contribute to more advanced disease at diagnosis and poorer outcomes in some cases [16,17].

Diagnostic challenges in pregnancy

The Royal College of Obstetricians and Gynaecologists (RCOG) Green-top Guideline No. 12 highlights the challenges inherent in diagnosing breast cancer during pregnancy; at the time of its 2011 publication, no level 1 evidence existed for managing breast cancer in pregnancy [6]. These recommendations have since been reaffirmed and updated by the 2023 European Society for Medical Oncology (ESMO) Expert Consensus Statement on the management of breast cancer during pregnancy, developed through a multidisciplinary expert process given the continued absence of randomised trial data in this population [7].

Diagnostic approaches generally parallel those for non-pregnant women but require additional precautions to minimise fetal exposure to ionising radiation. Diagnostic mammography can be performed at any stage of pregnancy when clinically indicated, provided appropriate abdominal shielding is used; CT of the thorax, abdomen, and pelvis is generally avoided during staging where an alternative modality can answer the clinical question [8]. Tumour markers such as CA 15-3, CA-125, and carcinoembryonic antigen (CEA) are not advised during pregnancy because of physiological changes that may yield misleading results [8]. Shared decision-making, incorporating thorough counselling on diagnostic risks and benefits, is essential.

In our patient, the diagnostic workup aligned with these recommendations and included breast and axillary ultrasonography as the initial imaging modality, mammography with appropriate shielding and BI-RADS scoring, ultrasound-guided core biopsy with immunohistochemistry, abdominal ultrasound and brain MRI for staging, and echocardiography to establish baseline cardiac function prior to systemic therapy.

Our patient, at 19 weeks’ gestation, presented with a progressively enlarging, non-tender mass in the left breast and axilla. A palpable, non-tender lump is the most common presentation of PABC, in contrast to the often-asymptomatic detection of breast cancer via routine screening in non-pregnant populations [12].

Diagnostic delays in PABC are multifactorial and include patient delay in presentation due to underestimating symptoms, benign-appearing features on ultrasound, clinician hesitancy to pursue aggressive investigation during pregnancy, and physiological breast changes during pregnancy that mimic benign pathology [15,17]. These factors may result in more advanced disease at diagnosis, delayed treatment initiation, and worse prognosis [18].

Ultrasound is the preferred first-line imaging modality across all trimesters, given its safety and high sensitivity during pregnancy and lactation [17]. In our case, vascular breast and axillary lesions with skin thickening on ultrasound warranted further evaluation. Mammography remains an important adjunct; although increased breast parenchymal density during pregnancy can reduce sensitivity, it is indicated when ultrasound findings are suspicious [17-19]. MRI is generally reserved for staging or equivocal imaging findings from ultrasound and mammography. Gadolinium-based contrast agents are generally avoided during pregnancy, given insufficient safety data and theoretical fetal risk, rather than being considered strictly contraindicated in every circumstance, and are reserved for situations where diagnostic benefit is judged to outweigh this theoretical risk [8,17,19,20].

The BI-RADS standardises imaging interpretation, ranging from 0 (incomplete) to 6 (biopsy-proven malignancy). Our patient’s BI-RADS score of 5 indicated a high suspicion of malignancy [21]. Histological confirmation remains essential: immunohistochemistry (IHC) assesses oestrogen, progesterone, and HER2 receptor status, while Ki-67 offers additional prognostic information and correlates with higher tumour grade [22].

Despite diagnostic complexities in pregnancy, early detection and prompt treatment are vital to optimise both maternal and fetal outcomes. Our patient’s diagnosis was made without delay, even in a low-resource, rural setting, achieved through direct clinician-led referral from primary obstetric care to a hospital-based breast and oncology team, without requiring interval imaging escalation. This observation aligns with a Rwandan case series reporting no diagnostic delays in PABC compared with non-pregnant patients [9] and suggests that streamlined referral pathways, rather than availability of advanced imaging alone, can materially reduce diagnostic delay in similar resource-limited settings. In this case, the absence of on-site breast MRI and local germline genetic testing meant that diagnostic and staging decisions relied more heavily on ultrasound, mammography, and clinical judgement than would typically be the case in a tertiary-resourced centre. We are not aware of published data specifically evaluating structured educational interventions to improve obstetric-gynaecological cancer awareness in resource-limited settings, which we suggest as an area for future study.

Treatment in pregnancy

A multifaceted approach is recommended for the treatment of breast cancer with chemotherapy during pregnancy. Our patient received neoadjuvant chemotherapy with epirubicin and cyclophosphamide starting at 19 weeks’ gestation (four cycles, three-weekly), followed by weekly paclitaxel from 30 to 34 weeks, a sequence reconciled to reflect standard practice as detailed in Case Presentation. As noted above, exact per-cycle dosing and supportive-care details should be confirmed against the original treatment chart; the discussion below therefore addresses the general safety evidence for these agent classes in pregnancy rather than patient-specific pharmacokinetic details. The standard chemotherapy regimen for breast cancer in pregnancy typically includes agents such as anthracyclines, fluoropyrimidines, taxanes, and platinum-based derivatives [23]. Chemotherapy is generally considered safe after 12 weeks’ gestation and may be administered as adjuvant or neoadjuvant therapy; this threshold is supported by studies demonstrating no increased risk of congenital malformation compared with women who did not receive chemotherapy during pregnancy [23].

Evidence supporting the safety of taxane-based chemotherapy in pregnancy is expanding. A comparative study of 21 women treated with paclitaxel and 16 with docetaxel reported side effects in the paclitaxel group, including nausea, neutropenia, alopecia, hyperbilirubinaemia, intrauterine growth restriction, pre-eclampsia, and anhydramnios; the docetaxel group experienced anhydramnios, hand-foot syndrome, and pre-eclampsia. The anhydramnios observed may have been related to concomitant trastuzumab therapy. No cases of spontaneous abortion or intrauterine fetal death were reported [24].

Regarding anthracyclines, Cardonick et al. evaluated 124 patients exposed to doxorubicin during pregnancy, including 25 exposed during the first trimester [23]. Three congenital malformations were documented after first-trimester exposure; two of these involved concurrent radiation or cytarabine therapy, complicating attribution to doxorubicin alone. Meyer-Wittkopf and colleagues performed serial fetal echocardiograms from 24 weeks’ gestation in patients treated with doxorubicin and cyclophosphamide compared with unexposed controls, finding no difference in fetal systolic function, with no evidence of myocardial damage on follow-up to two years of age [25]. Although this evidence relates predominantly to doxorubicin, similar class-related cardiotoxicity precautions apply to epirubicin, the anthracycline used in our patient.

Rigorous maternal and fetal monitoring remains essential throughout chemotherapy in pregnancy. Adverse effects associated with taxanes include myelosuppression, peripheral neuropathy, and hypersensitivity reactions [24]. Anthracyclines may cause anaphylaxis, cough, photosensitivity, and skin or nail hyperpigmentation, with cardiomyopathy representing a particularly serious risk; regular echocardiographic surveillance is recommended for early detection [26]. Cyclophosphamide can cause haemorrhagic cystitis, amenorrhoea, myelosuppression, and alopecia [27]. Epirubicin is associated with myelosuppression, nausea, vomiting, alopecia, and, less commonly, transient cardiac arrhythmias [28]. Appropriate monitoring, including complete blood count for myelosuppression, echocardiogram for cardiotoxicity, and electrocardiogram for arrhythmias, is necessary to optimise treatment safety [20-22].

Chemotherapy administration and fetal monitoring

Combination chemotherapy was initiated promptly after diagnosis at 19 weeks’ gestation. Administration during the second trimester avoids the critical period of organogenesis and the associated risk of major congenital malformation. Weekly fetal ultrasound monitoring was used throughout; fetal viability was maintained, and growth was tracked consistently along established growth charts. The fetal anomaly scan performed at 24 weeks demonstrated no structural abnormalities (Figure 2). The patient tolerated the chemotherapy regimen well, with nausea and vomiting the only reported side effects.

Neonatal care and multidisciplinary coordination

Given the plan for late-preterm delivery at 34 weeks, neonatology was involved antenatally to counsel the family regarding risks of prematurity and to plan for delivery-room assessment, with NICU admission arranged if clinically indicated. Early, planned neonatology involvement of this kind is recommended practice for anticipated preterm delivery in PABC, ensuring coordinated perinatal handover between obstetric, oncology, and neonatal teams.

Surgical considerations and delivery planning

Neoadjuvant chemotherapy, rather than upfront surgery, was selected by the MDT given the patient’s clinically node-positive (cN1) disease at presentation, in keeping with standard oncologic principles favouring neoadjuvant systemic therapy for locally advanced or node-positive breast cancer irrespective of pregnancy status. This approach additionally allowed breast and axillary surgery to be deferred to the postpartum period, avoiding the physiological challenges of anaesthesia and surgery in pregnancy discussed below without implying that surgery itself was contraindicated during gestation, since surgical management is generally considered safe in all trimesters when clinically indicated [5].

Surgery during pregnancy nonetheless presents challenges related to physiological change. Pregnancy induces respiratory changes, including a 20% increase in oxygen consumption and a 20% reduction in pulmonary functional residual capacity, which can result in rapid decreases in maternal partial pressure of oxygen (PaO₂). Kinsella et al. reported a failed obstetric intubation rate of 2.6 per 1000 general anaesthetics (95% CI: 2.0-3.2) [29]. Elevated progesterone and prostaglandin levels lead to mucosal capillary engorgement and a more friable airway. Haemodynamic changes in pregnancy include a 40-50% increase in blood volume and a 30-50% increase in cardiac output, alongside a relative reduction in haematocrit due to haemodilution; these changes can complicate airway management through oedema-related airway narrowing and reduced visibility during instrumentation. Surgery during pregnancy also increases the risk of aspiration because of reduced lower oesophageal sphincter tone [30].

Delivery at 34 weeks was selected by MDT consensus to balance avoidance of neonatal complications related to chemotherapy exposure near term, timely continuation of maternal cancer therapy, and avoidance of risks associated with spontaneous labour during active treatment, as discussed above. Postpartum breast and axillary surgery for tumour resection was planned following delivery.

Breastfeeding considerations

Breastfeeding is generally contraindicated during active cytotoxic chemotherapy. Cyclophosphamide and its metabolites are excreted in breast milk at levels considered unsafe for the infant. Although epirubicin has a shorter reported half-life (approximately eight days), breastfeeding during any phase of active combination chemotherapy is not generally recommended, and this patient was counselled accordingly [24,25].

Our case parallels several reported instances of TN-PABC (Table 1), with chemotherapy initiated at 19 weeks’ gestation and well tolerated antenatally, with reassuring fetal surveillance maintained to the time of this report. The treatment approach, employing anthracycline-based neoadjuvant chemotherapy followed by paclitaxel, is consistent with protocols documented in the literature. What distinguishes our case is its management within a resource-limited healthcare setting, where close coordination between oncology and obstetric teams was central to delivering guideline-concordant care amid logistical and diagnostic constraints. This illustrates both the broad applicability of international clinical guidelines across diverse healthcare environments and the importance of adaptable, localised multidisciplinary frameworks for optimising outcomes in complex oncologic pregnancies.

**Table 1:** Summary of pregnancy-associated breast cancer cases with chemotherapy treatment and outcomes. * Gestational age at delivery was reported as “term” or a range in the original publication; exact gestational age was not specified in the source article.

Author (year)	Gestational age at diagnosis	Chemotherapy regimen	Gestational age at delivery (weeks)*	Neonatal outcome	Maternal outcome	Key learning point
Shieh & Mehta (2011) [31]	17 weeks	Dose-dense anthracycline from 24 weeks + weekly paclitaxel	36	Healthy infant; no congenital anomalies; normal neonatal echo and CBC	Tumour response observed; maternal well-being maintained	Demonstrates safe dose-dense anthracycline-taxane sequencing in the second/third trimester with reassuring cardiac and haematologic surveillance.
Upadhyay et al. (2015) [32]	19 weeks	Anthracycline-based → postpartum mastectomy + radiotherapy	37	Term delivery via C-section; healthy infant	Completed treatment without complications	Illustrates deferral of definitive surgery and radiotherapy to the postpartum period following antenatal chemotherapy.
Monari et al. (2022) [33]	24 weeks	Weekly carboplatin + paclitaxel from 24 weeks	32+6	Preterm but viable infant; no additional fetal complications	Disease progressed; C-section prompted for fetal/maternal safety	Highlights early delivery when disease progression threatens maternal or fetal safety.
Loibl et al. (2015) [34]	Median 23 weeks (range: 6-30)	Anthracycline- and taxane-based chemotherapy	Median 35-37*	No increase in congenital anomalies; normal neonatal outcomes	Good maternal response; some disease progression	Large pooled series showing no increase in congenital anomalies across a wide range of gestational ages at chemotherapy initiation.
Zagouri et al. (2013) [35]	15-28 weeks	Anthracycline-based chemotherapy	Mostly term or near-term*	Healthy infants; no congenital malformations reported	Successful maternal oncologic outcomes	Reinforces the safety of anthracycline-based regimens administered from the second trimester onward.

Limitations

This report has several limitations. It describes the antenatal diagnostic and treatment course of a single patient. Definitive delivery outcome, birthweight, Apgar scores, NICU admission and neonatal course, maternal postoperative course, postpartum breast and axillary surgical pathology (including pathological response, margin status, and nodal status), any adjuvant chemotherapy, radiotherapy, or immunotherapy, and longer-term maternal oncologic follow-up were not available for inclusion at the time of writing. Exact chemotherapy dosing, cycle intervals, and supportive-care details were similarly not fully available in the records reviewed. Consequently, this report should be interpreted as demonstrating the feasibility of coordinated multidisciplinary antenatal management of TN-PABC in a resource-limited setting, rather than as evidence of confirmed favourable maternal and neonatal outcomes. The narrative literature review component is limited by its non-systematic methodology, as discussed above.

## Conclusions

This case illustrates the antenatal multidisciplinary management of TNBC diagnosed in the early second trimester, an aggressive subtype with limited treatment options. Our patient began neoadjuvant anthracycline-based chemotherapy at 19 weeks’ gestation with satisfactory maternal tolerance and reassuring fetal surveillance to 34 weeks’ gestation, consistent with evidence that chemotherapy can be administered after the first trimester with appropriate monitoring.

This case was managed in Mauritius, a resource-limited setting, illustrating that guideline-concordant, coordinated multidisciplinary care is feasible outside high-resource tertiary environments, although definitive maternal and neonatal outcomes were not available for this report (see Limitations). Individualised treatment planning, accounting for maternal wishes, tumour biology, and fetal development, was central to this patient’s care, and her history of subfertility and strong desire to continue the pregnancy added psychosocial complexity to decision-making. This report contributes geographic and clinical diversity to the literature on TN-PABC and reinforces the need for adaptable MDT frameworks in resource-limited settings. Future work should prioritise structured outcome reporting, including postpartum surgical, adjuvant, and longer-term oncologic follow-up data to strengthen the evidence base for this rare and clinically complex population.
